# Complete Genome Sequence of *Francisella* sp. Strain LA11-2445 (FDC406), a Novel *Francisella* Species Isolated from a Human Skin Lesion

**DOI:** 10.1128/MRA.01233-20

**Published:** 2021-01-14

**Authors:** Caroline Öhrman, Ingrid Uneklint, Linda Karlsson, Laurel Respicio-Kingry, Mats Forsman, Jeannine M. Petersen, Andreas Sjödin

**Affiliations:** a Swedish Defence Research Agency, CBRN Defence and Security, Umeå, Sweden; b Centers for Disease Control and Prevention, Division of Vector-Borne Diseases, Bacterial Diseases Branch, Fort Collins, Colorado, USA; Indiana University, Bloomington

## Abstract

Here, we present the 2,139,666-bp circular chromosome of *Francisella* sp. strain LA11-2445 (FDC406), a proposed novel species of *Francisella* that was isolated from a human cutaneous lesion and is related to *Francisella* species from marine environments.

## ANNOUNCEMENT

In 2011, a novel *Francisella* species was isolated from a lesion on the left ankle of a 69-year-old man in Louisiana (1). A bacterial culture swab taken from the underlying granular tissue yielded a pure isolate of a Gram-negative coccobacillus that grew on blood and chocolate agar. The isolate was initially suspected to be Francisella tularensis on the basis of growth characteristics, colony morphology, Gram staining, and biochemical testing; however, the results of F. tularensis-specific testing (direct fluorescent antibody and PCR assays) were negative. At the Centers for Disease Control and Prevention (CDC), the isolate, designated LA11-2445, was identified as a novel *Francisella* species by multilocus sequence comparison, with the greatest nucleotide similarities to *Francisella* organisms associated with marine environments (1).

**FIG 1:**
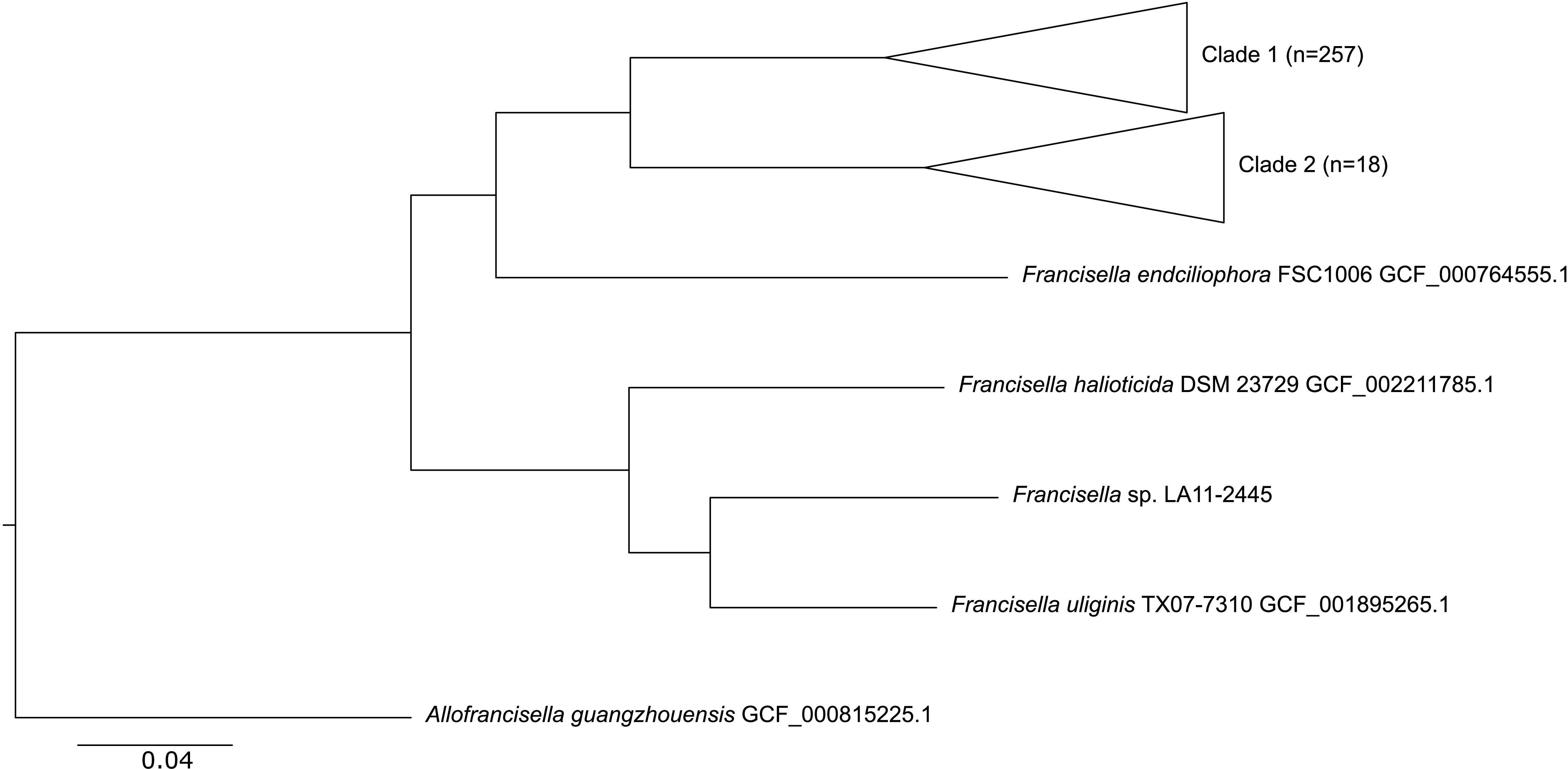
Whole-genome NJ phylogeny with 280 genomes, showing the relation of LA11-2445 to 279 other genomes within the genus *Francisella*. The nomenclature of clades 1 and 2 is from a previous publication (21). Public genomes are labeled using the RefSeq assembly accession number used.

The isolate was grown on cysteine heart agar with 9% chocolatized sheep blood for 48 h at 35°C in an ambient atmosphere. DNA extracted using the QIAamp DNA minikit (Qiagen, Hilden, Germany) was sent from the CDC to the Swedish Defense Research Agency and designated collection number FDC406. A short-read library was prepared with the Nextera XT kit (Illumina, San Diego, CA, USA) and sequenced on an Illumina MiSeq system (500 cycles), generating 1,354,128 reads with an *N*_50_ value of 251 bp. For long-read sequencing, the DNA was amplified with the multiple-displacement amplification (MDA) REPLI-g midikit (Qiagen), and a library was prepared with a 1D ligation sequencing kit (SQK-LSK108) with no shearing or size selection, barcoded with a 1D native barcoding kit (EXP-NBD103), and sequenced with an Oxford Nanopore Technologies (Oxford, UK) MinION system (R9.4). Nanopore reads were demultiplexed using Albacore v2.1.3 (Oxford Nanopore Technologies), generating 63,244 reads with an *N*_50_ value of 5,027 bp. Illumina reads were trimmed using Trimmomatic v0.38 (2) and Nanopore reads using Porechop v.0.2.3_seqan2.1.1 (3); quality was ensured with FastQC v0.11.9 (4) and NanoPlot v1.20.0 (5). A one-sequence complete circular chromosome was generated with long and short reads as inputs using the hybrid assembler Unicycler v0.4.7 with default settings (6), including two rounds of polishing with Pilon v1.23 (7) and rotation to *dnaA* as the start at position 463527. No incongruences were found in the assembly when it was compared, using DNAdiff v1.3 (8), with a short-read assembly generated with ABySS v2.1.4 (9). To verify circularization and to find possible scaffolding errors, Nanopore reads were mapped to the assembly using minimap2 v2.15 (10), followed by variation calling using Sniffles v1.0.10 (11). The scaffold ends were spanned by >10× coverage, and no incongruences were found. The genome was annotated with NCBI Prokaryotic Genome Annotation Pipeline (PGAP) v4.11 (12, 13). A total of 279 genomes of the genus *Francisella* according to GTDB taxonomy (14), rooted using one genome of the nearest neighbor *Allofrancisella* (GenBank accession number GCF_000815225.1), were pairwise aligned to the reference strain SCHUS4 (GenBank accession number GCF_000008985.1) using progressiveMauve vsnapshot_2015_02_13 (15, 16). A whole-genome neighbor-joining (NJ) phylogenetic tree, using a Jukes-Cantor model, was calculated with FastTree v2.1.10 (17, 18) and visualized with FigTree v1.4.3 (19) (Fig. 1). The average nucleotide identity (ANI) was calculated with pyani v0.2.10 (20). All software was executed using default settings unless otherwise stated. This work was approved by the institutional review board at the CDC.

The *Francisella* sp. LA11-2445 circular chromosome is 2,139,666 bp in size, has a GC content of 31.84%, and consists of 1,978 protein-coding sequences. The phylogeny is in agreement with that in the case report (1) (Fig. 1). An ANI of 83.8% with respect to the closest neighbor, TX07-7310, an isolate from seawater from the Gulf of Mexico, is consistent with LA11-2445 being a novel species of *Francisella*.

### Data availability.

The complete genome sequence for LA11-2445 (FDC406) is the first version and has been deposited in GenBank under the accession number CP041030, and the reads have been deposited in the SRA under accession numbers SRR11853262 and SRR11853263.
